# Blood pressure and intraocular pressure in the Canadian Longitudinal Study on Aging: a bidirectional Mendelian randomization analysis

**DOI:** 10.3389/fgene.2026.1967280

**Published:** 2026-10-21

**Authors:** Joseph Bastasic, Mohan Rakesh, Ellen E. Freeman, Julian Little, Kelly M. Burkett, Marie-Hélène Roy-Gagnon

**Affiliations:** 1 School of Epidemiology and Public Health, University of Ottawa, Ottawa, ON, Canada; 2 Department of Mathematics and Statistics, University of Ottawa, Ottawa, ON, Canada

**Keywords:** CLSA, gene-environment interaction, glaucoma, intraocular pressure, Mendelian randomization, blood pressure

## Abstract

**Purpose:**

To investigate the association of blood pressure (BP) with intraocular pressure (IOP), determine whether these associations are modified by genetic susceptibility to elevated IOP, and assess potential causality using Mendelian randomization (MR).

**Methods:**

Cross-sectional analyses were conducted using baseline data from the Canadian Longitudinal Study on Aging (CLSA) Comprehensive Cohort. Non-closely related participants of European descent with complete data for IOP and BP were eligible for individual-level data analyses (*N* = 21,633). Associations of systolic BP (SBP), diastolic BP (DBP) and pulse pressure (PP) with corneal-compensated IOP were assessed using multivariable linear regression. An IOP polygenic risk score (PRS) was used to measure genetic susceptibility to elevated IOP. One-sample and two-sample MR analyses were conducted to evaluate potential causality.

**Results:**

Multivariable linear regression found SBP, DBP, and PP to be positively associated with IOP after adjustment for relevant covariates (*P* ≤ 3.82 × 10^−17^). Interaction analyses did not find the IOP PRS to influence these associations (*P* ≥ 0.122). One-sample MR found genetically predicted SBP to be positively associated with IOP (*β* = 0.227 mm Hg per 10 mmHg increase in SBP, 95% confidence interval (CI) [0.074, 0.381], *P* = 0.004). Reverse one-sample MR did not provide evidence of IOP having a causal effect on BP (*P* ≥ 0.103). Similar results for the observational and bidirectional one-sample MR analyses were obtained after excluding participants taking IOP-lowering or antihypertensive medications. Two-sample MR provided some evidence of a positive causal effect of SBP on IOP. Significant positive associations were observed using the inverse-variance weighted multiplicative random-effects (IVW-MRE; *β* = 0.216 mmHg per 10 mmHg increase in SBP, 95% CI [0.043, 0.390], *P* = 0.015), MR contamination mixture (MR-ConMix; *β* = 0.314 mmHg per 10 mmHg increase in SBP, 95% CI [0.134, 0.565], *P* = 0.001), and MR Pleiotropy RESidual Sum and Outlier (MR-PRESSO; *β* = 0.239 mmHg per 10 mmHg increase in SBP, 95% CI [0.075, 0.402], *P* = 0.004) methods. However, there was evidence of directional pleiotropy based on the MR-Egger intercept test (*P* = 0.049). Similar two-sample MR results were obtained using IOP summary statistics from that CLSA after excluding participants taking IOP-lowering medications, although the MR-Egger intercept test was non-significant (*P* = 0.062). Analyses using Steiger-filtered genetic instruments provided some support for the primary two-sample MR results, but not the primary one-sample MR results.

**Conclusion:**

SBP, DBP, and PP are positively associated with IOP and genetic susceptibility to elevated IOP does not appear to modify these associations. One-sample and two-sample MR analyses provided some evidence of a potential causal effect of SBP on IOP. If confirmed, a causal effect of BP on IOP could lead ophthalmologists treating elevated IOP to encourage their patients to also have their BP under control.

## 1 Introduction

Elevated intraocular pressure (IOP) is the only known modifiable risk factor for glaucoma, a multifactorial eye disease that is typically asymptomatic and can lead to irreversible blindness (Weinreb et al., 2014). The global number of glaucoma cases among individuals 40–80 years old is predicted to increase from 76.0 million in 2020 to 111.8 million 2040 (Tham et al., 2014). The most common type of glaucoma is primary open-angle glaucoma (POAG), which is idiopathic and defined by an unobstructed iridocorneal angle in the eye (Quigley and Broman, 2006; Tham et al., 2014; Weinreb et al., 2014). IOP is a polygenic trait (Gao et al., 2018; Khawaja et al., 2018; MacGregor et al., 2018) and genetic predisposition to elevated IOP has been shown to be predictive of POAG risk and severity (Khawaja et al., 2018; MacGregor et al., 2018; Gao et al., 2019; Qassim et al., 2020). Regardless of glaucoma subtype, lowering IOP is currently the only effective method for impeding disease progression (Stein et al., 2021).

For decades, researchers have sought to understand how blood pressure (BP) is related to IOP (Leeman and Kestelyn, 2019). Population-based studies have generally found systolic BP (SBP), diastolic BP (DBP), and systemic hypertension to be positively associated with IOP (Bae et al., 2014; Zhao et al., 2014; Chan et al., 2016; Han et al., 2018; Yasukawa et al., 2022). A meta-analysis by Zhao et al. (2014) estimated IOP to increase on average by 0.26 mmHg (95% confidence interval (CI) [0.23, 0.28]) per 10 mmHg increase in SBP and by 0.17 mmHg (95% CI [0.11, 0.23]) per 5 mmHg increase in DBP. The SBP estimate was based on 13 cross-sectional and four longitudinal analyses, while the DBP estimate was based on ten cross-sectional and four longitudinal analyses. Both acute and long-term reductions in BP have been observed to be associated with decreased IOP (Klein et al., 2005; Tiambeng et al., 2022). Pulse pressure (PP), calculated as the difference between SBP and DBP and an indirect measure of arterial stiffness and vascular health (Dart and Kingwell, 2001), has also been found to be positively associated with IOP (Plotnikov et al., 2022; Vallée et al., 2025). It has been hypothesized that BP could affect IOP by altering the production and/or drainage of aqueous humor (Bulpitt et al., 1975; Shiose, 1984).

The relationship between BP and glaucoma appears to be more complex. There is evidence to suggest that both low and high BP are associated with an increased risk of the disease (Leske et al., 2008; Memarzadeh et al., 2010; Zhao et al., 2014; Chung et al., 2015; Tham et al., 2018; Kim and Choi, 2019). The vascular theory of glaucoma argues that glaucoma is caused by ischemic damage to the optic nerve, due to an insufficient blood supply (Wang X. et al., 2023). Biological mechanisms have been proposed to explain how systemic hypertension could indirectly cause a decrease in ocular perfusion pressure, which would result in reduced blood flow to the optic nerve (Leeman and Kestelyn, 2019; Nislawati et al., 2021).

To our knowledge, only a few studies have used Mendelian randomization (MR) to investigate whether BP has a causal effect on IOP or glaucoma (Liu et al., 2022; Plotnikov et al., 2022; Tan et al., 2022; Julian et al., 2023; Wang K. et al., 2023; Rajasundaram et al., 2024). Unlike traditional observational analyses, MR uses genetic variants as instrumental variables (IVs) to address potential confounding and reverse causation (Lawlor et al., 2008). MR involves estimating the associations between genetic instruments and both the exposure and the outcome. These associations can be estimated within a single cohort (one-sample MR) or in separate cohorts (two-sample MR) of the same underlying population (Boehm and Zhou, 2022). All previously identified MR studies examining the relationships between BP, IOP, and glaucoma used two-sample MR designs based on genome-wide association study (GWAS) summary statistics (Liu et al., 2022; Plotnikov et al., 2022; Tan et al., 2022; Julian et al., 2023; Wang K. et al., 2023; Rajasundaram et al., 2024). Only one study additionally incorporated a one-sample MR analysis, which was used to evaluate a potential non-linear causal relationship between BP and IOP (Plotnikov et al., 2022).

Previous MR studies have reported inconsistent findings regarding the causal effects of BP on IOP and glaucoma. Among the three MR studies evaluating IOP as an outcome, two reported positive associations of genetically predicted SBP and DBP with IOP (Plotnikov et al., 2022; Rajasundaram et al., 2024), whereas the third found no significant associations for either genetically predicted SBP or DBP (Tan et al., 2022). For glaucoma, two MR studies reported genetically predicted SBP to be positively associated with POAG (Tan et al., 2022; Wang K. et al., 2023), while the other studies did not find strong evidence for a causal relationship between BP and glaucoma (Liu et al., 2022; Plotnikov et al., 2022; Julian et al., 2023; Rajasundaram et al., 2024). The only study evaluating genetically predicted PP found no significant association with either IOP or glaucoma (Plotnikov et al., 2022).

The goal of the current study was to investigate the relationship between BP and IOP among study participants in the Canadian Longitudinal Study on Aging (CLSA). The association was first examined using multivariable linear regression models with adjustment for relevant confounders. One-sample and two-sample MR analyses were conducted to assess potential causality. Unlike previous MR studies of BP and IOP (Plotnikov et al., 2022; Tan et al., 2022; Rajasundaram et al., 2024), which relied primarily on summary-level data, this study made greater use of individual-level data, allowing for improved covariate control and more flexible MR analyses (Davies et al., 2018). An IOP polygenic risk score (PRS), constructed using independently associated single nucleotide polymorphisms (SNPs) identified through conditional analysis (Khawaja et al., 2018), was used to assess whether genetic susceptibility to elevated IOP modified the association between BP and IOP.

## 2 Methods

### 2.1 Study population and design

The CLSA Comprehensive Cohort (*N* = 30,097) consists of community-dwelling adults aged 45–85 years from seven Canadian provinces (Alberta, British Columbia, Manitoba, Newfoundland and Labrador, Nova Scotia, Ontario, and Quebec) (Raina et al., 2009; Raina et al., 2019). Recruitment and baseline data collection lasted from 2010 to 2015. Provincial healthcare databases and random digit dialing were used to recruit individuals living within 25–50 km of one of 11 CLSA data collection sites for this cohort. Individuals living in Canadian territories, Federal First Nations reserves and similar provincial settlements were excluded, as were those who were cognitively impaired at baseline, unable to speak English or French, institutionalized at baseline, or full-time members of the Canadian Armed Forces. Participants completed a detailed questionnaire at home and visited data collection sites to answer additional questionnaires, undergo physical assessments, and provide non-fasting blood and urine samples. All questionnaires were administered through in-person interviews. Written informed consent was obtained from all participants. Research ethics board approval was obtained for all CLSA affiliated sites in July 2010. The University of Ottawa provided research ethics board approval for our analysis in December 2018.

### 2.2 Intraocular pressure

Tonometry testing at CLSA data collection sites was performed using the Reichart Ocular Response Analyzer (Reichert Technologies, Depew, NY, United States). Consistent with previous studies (Khawaja et al., 2018; Grant et al., 2023; Grant et al., 2024), the average corneal-compensated IOP of both eyes was used for analysis. The IOP of the right and left eyes was moderately correlated (*r* = 0.57). IOP measurements from a single eye were used when data was missing for the other eye. Study participants with a detached retina or those who had undergone eye surgery within threemonths prior to the ophthalmic examination were excluded from having their IOP measured. Individuals with an eye infection in both eyes at the time of the examination were also ineligible. Pretreatment IOP for those taking any IOP lowering eye drops was estimated by dividing their mean IOP by 0.7, to account for the average treatment effect of these medications (Van der Valk et al., 2005; Khawaja et al., 2018). Participants using any IOP-lowering eye drops were identified through a search of reported medications by Drug Identification Numbers (DINs). Mean IOP observations (corrected or uncorrected) exceeding 60 mmHg were deemed to be likely caused by measurement error and were excluded from the study (*n* = 3).

### 2.3 Blood pressure

BP was measured six times by trained staff using the BpTru BPM-200 automated BP monitor (BpTRU Medical Devices, Coquitlam, BC, Canada). Mean SBP and DBP were calculated from five measurements, excluding the first. In accordance with other studies (Warren et al., 2017; Evangelou et al., 2018; Keaton et al., 2024), participants who reported to be currently taking any antihypertensive medications had 15 mmHg and 10 mmHg added to their mean SBP and DBP, respectively (Tobin et al., 2005). PP was calculated as the difference between mean SBP and DBP. Unless otherwise specified, the imputed pretreatment mean SBP and DBP values were used to calculate PP and for all subsequent analyses.

### 2.4 Demographic, lifestyle, health, and serum lipid biomarker data

The covariate data used in our analysis were primarily obtained from questionnaires administered through in-person interviews. Demographic data collected through the at-home questionnaire included information on age, sex, marital status, education, and annual household income. Lifestyle data, such as smoking status and alcohol intake frequency, were also collected by this questionnaire. Diabetes status was determined based on responses to the questionnaires completed at data collection sites, which focused on identifying health comorbidities.

Smoking status and alcohol intake frequency were defined using responses to questions on lifetime and current habits. Never smokers were defined as participants who reported to have not smoked at least 100 cigarettes during their lifetime. For those who had smoked at least 100 cigarettes during their lifetime, their smoking habits during the 30 days prior to being interviewed were used to classify them as either current or former smokers. Those considered to be occasional or daily smokers during this period were defined as current smokers, while those who reported to have not smoked during this period were defined as former smokers. Regarding alcohol intake frequency, never drinkers were participants who reported to have never had any alcohol. Based on drinking habits during the 12 months before the interview, participants who had consumed alcohol during their lifetime were classified as occasional (0–3 times a month), weekly (1–5 times a week), or daily (≥6 times per week) drinkers.

Body mass index (BMI) and serum lipid biomarkers were also used as covariates. Weight (kg) and standing height (m) were measured using the 140–10 Healthweigh Digital Physician Scale (Rice Lake Weighing Systems, Rice Lake, WI, United States) and the Seca 213 stadiometer (seca GmbH and Co. KG., Hamberg, Germany), respectively. Two measurements were taken for each trait, and average values were used to calculate BMI (kg/m^2^). Serum levels (mmol/L) of high-density lipoprotein cholesterol (HDL-C), total cholesterol (TC), and triglycerides (TG) were measured from non-fasting blood samples using the cobas 8,000 modular analyzer series (Roche Diagnostics, Indianapolis, IN, United States). A natural logarithmic transformation was applied to the TG variable (log-TG), since the distribution of serum TG in the study population was heavily skewed to the right.

### 2.5 Genetic data

The third release of the CLSA genomic data has whole-genome genotyping and imputed data for 26,622 study participants from the CLSA Comprehensive Cohort (Forgetta et al., 2022). The Affymetrix UK Biobank Axiom array was used for genotyping 749,409 known genetic markers (genome build GRCh37/hg19). The CLSA performed extensive quality control assessments of genotyped markers and samples. Marker-based quality control testing examined Hardy-Weinberg equilibrium, genotype concordance among control sample replicates, and the consistency of genotype frequencies across batches and between sexes. Sample-based quality control testing was conducted to identify potential low-quality samples (excessive missing genotype data and/or heterozygosity), assess familial relatedness, and investigate genetic ancestry. The CLSA used KING (version 2.1.3) software (Manichaikul et al., 2010) to calculate pairwise kinship coefficients and infer relatedness among participants with genotype data. Principal component analysis found 92.6% of study participants with genomic data (*n* = 24,655) to be of European descent. All non-closely related participants of European descent who passed sample quality control criteria were eligible for analysis. Participants not flagged by the CLSA as having an inferred third-degree or closer relationship with another participant (Forgetta et al., 2022) were considered non-closely related. Figure 1 provides an overview of the selection process for this study. Through the University of Michigan Imputation Service (Das et al., 2016), the CLSA performed genotype imputation using the TOPMed r2 reference panel, which consists of 308,107,085 genetic markers and was developed using 97,256 reference samples (Taliun et al., 2021).

**FIGURE 1:**
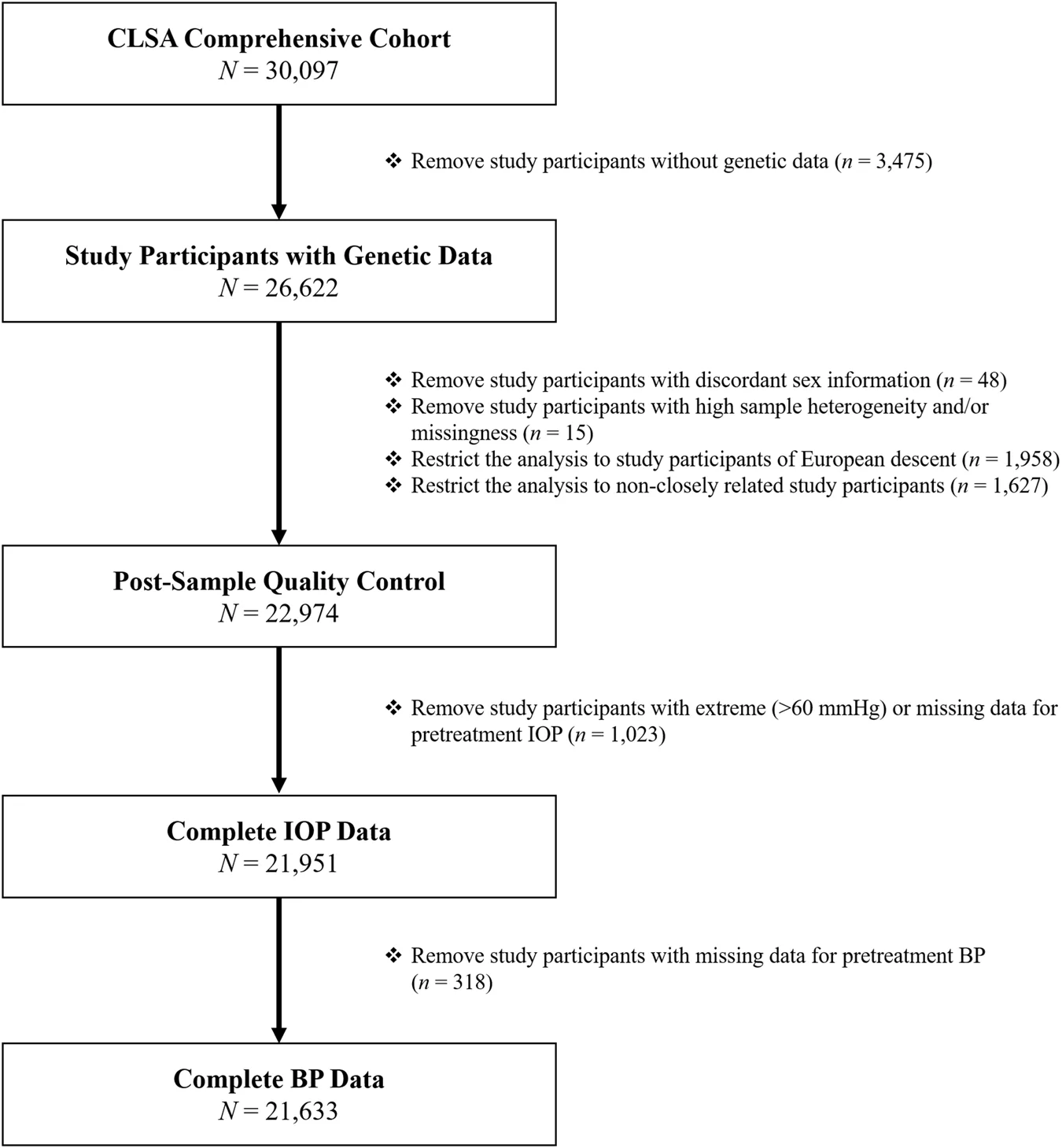
Overview of the sample selection process. BP, blood pressure; CLSA, Canadian Longitudinal Study on Aging; IOP, intraocular pressure.

The current study used the imputed genotype data from the CLSA (genome build GRCh38/hg38). Data on allelic dosages for required SNPs was extracted from BGEN files using bgenix (version 1.1.7, revision) (Band and Marchini, 2018) and PLINK (v2.00a6) (Purcell et al., 2007; Chang et al., 2015). Data harmonization involved removing SNPs with inconsistent allele assignments between datasets, SNPs with strand orientations that could not be inferred by allele frequency, and SNPs with a batch imputation quality score (INFO) ≤ 0.75 in the CLSA imputed genotype data. Palindromic SNPs with a minor allele frequency (MAF) > 0.4 (regardless of the dataset) were considered ambiguous and excluded from the analysis. All palindromic SNPs were excluded if the base data did not provide information on allele frequencies (Collister et al., 2022). SNP genomic coordinates reported in genome build GRCh37/hg19 were converted to GRCh38/hg38 using dbSNP Build 157 (Sherry et al., 1999; Sherry et al., 2001). SNP rsIDs were first verified or updated in GRCh37/hg19 prior to retrieving corresponding positions in GRCh38/hg38. SNPs with unresolved discrepancies in reported rsIDs or genomic coordinates were removed from the analysis.

### 2.6 Core instrumental variable assumptions

For a valid MR analysis, it is imperative that genetic instruments are strongly associated with the exposure of interest, independent of confounders, and affect the outcome only through the exposure. These conditions, collectively referred to as the core IV assumptions, correspond to the relevance, independence, and exclusion-restriction assumptions, respectively (Lawlor et al., 2008). While the relevance assumption can be directly tested by measuring instrument strength (Staiger and Stock, 1997; Burgess et al., 2011), it is not possible to completely verify the other core IV assumptions. Analytical strategies and robust MR methods have been developed to identify and address potential violations of the independence and exclusion-restriction assumptions (Davies et al., 2018; Skrivankova et al., 2021). Associations between genetic instruments and measured confounders can be examined as a means of identifying blatant violations of the independence assumption. Potential confounding by population stratification and familial effects can be minimized by restricting analyses to non-closely related participants of a homogeneous ancestry and adjusting for genetic principal components (Carter and Anderson, 2024). Robust MR methods rely on alternative assumptions for obtaining consistent causal effect estimates when analyses include invalid genetic instruments (Slob and Burgess, 2020; Hu et al., 2024).

Common robust methods for two-sample MR analyses include the MR-Egger (Bowden et al., 2015), weighted median (Bowden et al., 2016a), weighted mode (Hartwig et al., 2017), MR contamination mixture (MR-ConMix) (Burgess et al., 2020), and MR Pleiotropy RESidual Sum and Outlier (MR-PRESSO) (Verbanck et al., 2018) methods. The MR-Egger method allows for directional pleiotropy, provided that the direct effects of genetic instruments on the outcome are independent of their effects on the exposure. This condition is known as the Instrument Strength Independent of Direct Effect (InSIDE) assumption (Bowden et al., 2015). The weighted median method provides consistent causal effect estimates when at least 50% of the analytical weight is contributed by valid genetic instruments (majority valid assumption) (Bowden et al., 2016a). The weighted mode method provides consistent causal effect estimates when the largest proportion of the analytical weight is contributed by a single cluster of valid genetic instruments with similar causal effect estimates (plurality valid assumption) (Hartwig et al., 2017). The MR-ConMix method models genetic variants as a mixture of valid and invalid genetic instruments. It provides consistent causal effect estimates when the valid genetic instruments constitute the largest mixture component (plurality valid assumption) (Burgess et al., 2020). MR-PRESSO identifies and removes outlying variant-specific causal effect estimates. Correct identification of outliers requires that more than 50% of variants be valid genetic instruments (majority valid assumption) (Verbanck et al., 2018).

### 2.7 Genetic instruments

The largest available single-stage GWAS meta-analysis of BP (Keaton et al., 2024) was used to select genetic instruments for SBP, DBP, and PP (Supplementary Table S1). This analysis included up to 1,028,980 individuals of European descent. The per-variant sample sizes ranged from 451,315 to 1,017,460 for SBP, 455,715 to 1,028,980 for DBP, and 448,695 to 1,017,100 for PP. The study identified 2,103 independent genetic loci associated with at least one of the three BP traits. For each trait, independent SNPs achieving genome-wide significance (*P* < 5 × 10^−8^) were identified using LD clumping and selected as genetic instruments. LD clumping (*r* ^2^ < 0.001 and 10,000 kb window) was performed using the 1000 Genomes Project Phase 3 European LD reference panel (*n* = 503) (Auton et al., 2015). Initially, 545, 513, and 483 SNPs were identified as candidate genetic instruments for SBP, DBP, and PP, respectively. Of these, 541, 507, and 478 SNPs, respectively, were available in the CLSA imputed genotype data. Given that ≤1.2% of SNPs were missing for each BP trait, the use of LD proxy SNPs was deemed unnecessary. After harmonization, a total of 522, 477, and 456 SNPs were retained as instruments for SBP, DBP, and PP, respectively.

Genetic instruments for IOP were selected from an IOP GWAS meta-analysis conducted by Khawaja et al. (2018) in 139,555 individuals of European descent (Supplementary Table S1). A conditional analysis identified 133 SNPs independently associated with IOP. The CLSA imputed genotype data included 130 (97.7%) of these SNPs. After harmonization, a total of 122 (91.7%) of the original 133 SNPs were retained as genetic instruments for IOP.

Externally weighted PRSs for SBP, DBP, PP, and IOP were constructed using the CLSA imputed genotype data. These PRSs were calculated by taking a weighted sum of allelic dosages: $PRS=\sum_{i=1}^{N}{\hat{\beta }}_{i}\times G_{i}$ , where ${\hat{\beta }}_{i}$ is the estimated effect size for the $i^{th}$ SNP (with respect to the trait-increasing effect allele), $G_{i}$ is the allelic dosage for the trait-increasing effect allele, and N is the number of SNPs used for the PRS calculation. One-sample MR analyses were conducted using these PRSs as genetic instruments (Burgess and Thompson, 2013). Forward one-sample MR analyses used the SBP, DBP, and PP PRS instruments. The IOP PRS was used for gene-environment (G × E) interaction analyses and as the genetic instrument for reverse one-sample MR analyses. All PRSs were standardized to have a mean of 0 and a standard deviation (SD) of 1 for analyses.

### 2.8 Statistical analysis

Baseline characteristics of participants eligible for individual-level data analyses were summarized as mean ± SD for continuous variables and *n* (%) for categorical variables. The association of BP with IOP was investigated using multivariable linear regression models adjusted for age, sex, province, marital status, education, annual household income, diabetes, smoking status, alcohol intake frequency, BMI, HDL-C, TC, log-TG, and the top ten genetic principal components (*N* = 21,125). Assessment of potential G × E interactions between BP and genetic susceptibility to elevated IOP involved fitting models that included the IOP PRS and testing the statistical significance of interaction terms. Results from linear regression models with minimal covariate adjustment (age, sex, province, and the top ten genetic principal components) were reported for comparison (*N* = 21,633). As part of a sensitivity analysis, the BP-IOP association and G × E interaction analyses were repeated after excluding participants taking IOP-lowering or antihypertensive medications.

The validity of the PRS instruments was evaluated before conducting the bidirectional one-sample MR analyses. Instrument strength (IV relevance assumption) was assessed using partial *F*-statistics obtained by regressing SBP, DBP, PP, or IOP on their corresponding PRS. Genetic instruments with an *F*-statistic >10 were considered strong instruments at low risk for weak instrument bias (Staiger and Stock, 1997; Burgess et al., 2011). Partial *R* ^2^ values were also calculated to quantify the proportion of residual variance explained by each PRS instrument. Potential violations of the IV independence assumption were assessed by regressing measured confounders on each PRS instrument (Carter and Anderson, 2024). Linear regression was used for continuous confounders, whereas binary and multinomial logistic regression were used for categorical confounders. For multicategory confounders, overall associations were assessed using likelihood ratio tests comparing models with and without the PRS instrument. PRS-confounder associations with *P* < 0.005 (*α*_*Bonferroni*_ = 0.05/10) were considered non-spurious. All models testing IV validity were adjusted for age, sex, province, and the top ten genetic principal components.

One-sample MR analyses were conducted using two-stage least squares (2SLS) regression (Boehm and Zhou, 2022). This method involves two sequential regressions: (1) regressing each exposure on its respective PRS instrument and relevant covariates, followed by (2) regressing the outcome on the predicted exposure values and the same covariates. For N study participants (indexed by i=1,...,N ), let $X_{i}$ denote a continuous exposure, $Y_{i}$ a continuous outcome, $G_{i}$ a continuous genetic instrument, and $C_{i}$ a vector of measured covariates. The 2SLS regression method with a single continuous genetic instrument can be expressed by the following equations:

$$X_{i}=\gamma_{0}+\gamma_{1}G_{i}+\gamma_{2}C_{i}+\epsilon_{Xi}$$ (1)

$$Y_{i}=\beta_{0}+\beta_{1}{\hat{X}}_{i}+\beta_{2}C_{i}+\epsilon_{Yi}$$ (2)

where ${\hat{X}}_{i}$ in Equation 2 is the genetically predicted value of the exposure obtained from Equation 1. The error terms for Equations 1, 2 are represented by $\epsilon_{Xi}$ and $\epsilon_{Yi}$ , respectively. The $\beta_{1}$ coefficient from Equation 2 represents the causal effect of the exposure.

In the primary analyses, one-sample MR models were adjusted for age, sex, province, and the top ten genetic principal components (*N* = 21,633). Analyses were repeated with adjustment for the complete set of covariates included in the fully adjusted multivariable linear regression models (*N* = 21,125) to assess the robustness of the findings to additional covariate adjustment. As in the observational analyses, one-sample MR analyses were repeated after excluding participants taking IOP-lowering or antihypertensive medications.

The same SNP-BP effect estimates from the BP GWAS meta-analysis (Keaton et al., 2024) used to construct the BP PRS instruments were also used in the two-sample MR analyses. Corresponding SNP-IOP association estimates were obtained from the CLSA data (*N* = 21,951) using multivariable linear regression adjusted for age, sex, province, and the top ten genetic principal components. The primary two-sample MR analysis was conducted using the inverse-variance weighted (IVW) estimator (Burgess et al., 2013). An IVW fixed-effects (IVW-FE) model was used in the absence of significant between-SNP heterogeneity, whereas an IVW multiplicative random-effects (IVW-MRE) model was used when there was significant heterogeneity (Cochran’s *Q* test *P*-value <0.05) (Burgess et al., 2013; Bowden et al., 2017; Bowden and Holmes, 2019). The IVW estimator is the most statistically efficient two-sample MR estimator and provides unbiased causal effect estimates when all genetic instruments satisfy the core IV assumptions (Burgess et al., 2013; Bowden et al., 2015; Bowden et al., 2016b; Bowden et al., 2017; Slob and Burgess, 2020).

The MR-Egger (Bowden et al., 2015), weighted median (Bowden et al., 2016a), weighted mode (Hartwig et al., 2017), MR-ConMix (Burgess et al., 2020), and MR-PRESSO (Verbanck et al., 2018) methods were performed as sensitivity analyses. These robust MR methods can handle different types of violations of the core IV assumptions by relying on alternative assumptions (Bowden et al., 2015; Bowden et al., 2016a; Hartwig et al., 2017; Verbanck et al., 2018; Burgess et al., 2020; Slob and Burgess, 2020; Hu et al., 2024). Heterogeneity in SNP-specific causal estimates was assessed using Cochran’s *Q* statistic (Bowden and Holmes, 2019) and quantified using the *I* ^2^ statistic (Higgins et al., 2003). Rücker’s *Q*′ statistic was used to assess residual heterogeneity after applying the MR-Egger method to account for potential directional pleiotropy (Bowden and Holmes, 2019). MR-Egger analyses using genetic instruments with $I_{GX}^{2}$ values >90% were considered at low risk for substantial regression dilution bias (Bowden et al., 2016b). Directional and overall horizontal pleiotropy were assessed by the MR-Egger intercept test and the MR-PRESSO global test, respectively (Bowden et al., 2015; Verbanck et al., 2018). The proportion of SNPs identified as valid genetic instruments by MR-ConMix was also reported (Burgess et al., 2020).

All analyses were performed in Posit Team (2026) RStudio version 2026.08.2+200 (Posit Software, PBC, Boston, MA, United States) using R 4.5.3 (R Foundation for Statistical Computing, Vienna, Austria). The 2SLS regression models for one-sample MR were fitted using the *ivreg* (version 0.6–7) R package. The two-sample MR analyses were conducted using the *TwoSampleMR* (version 0.7.4) (Hemani et al., 2018), *MendelianRandomization* (version 0.10.0) (Yavorska and Burgess, 2017; Broadbent et al., 2020; Patel et al., 2023), and *MRPRESSO* (version 1.0) (Verbanck et al., 2018) R packages. Bonferroni correction was applied where appropriate to account for multiple testing. The primary one-sample and two-sample MR analyses were repeated after using Steiger filtering to exclude BP associated SNPs that explained a greater proportion of variance in the outcome than the exposure (Hemani et al., 2017). For the one-sample MR analyses, this involved recalculating the externally weighted BP PRS instruments using the SNPs retained after Steiger filtering. The primary two-sample MR analyses were also repeated using SNP-IOP association estimates from the CLSA that excluded participants taking IOP-lowering medications.

## 3 Results

### 3.1 Descriptive characteristics

A total of 21,633 participants of European descent (71.9% of the CLSA Comprehensive Cohort) with genotype data passed sample quality control criteria and had complete data for BP exposures and IOP. Table 1 summarizes baseline characteristics of these participants.

**TABLE 1:** Baseline characteristics of CLSA comprehensive cohort participants.

Characteristic	Total (*N* = 21,633)
SBP (mmHg)	125.4 ± 19.3
DBP (mmHg)	76.9 ± 11.0
PP (mmHg)	48.5 ± 13.7
Antihypertensive medication	
No	15,392 (71.2)
Yes	6,241 (28.8)
IOP (mmHg)	16.3 ± 3.9
IOP‐lowering medication	
No	21,165 (97.8)
Yes	468 (2.2)
Age (years)	62.9 ± 10.1
Sex	
Male	10,809 (50.0)
Female	10,824 (50.0)
Province	
Alberta	2,154 (10.0)
British Columbia	4,424 (20.5)
Manitoba	2,332 (10.8)
Newfoundland and labrador	1,546 (7.1)
Nova Scotia	2,255 (10.4)
Ontario	4,906 (22.7)
Quebec	4,016 (18.6)
Marital status	
Single	6,555 (30.3)
Partnered	15,074 (69.7)
Education	
More than Bachelor’s	4,596 (21.3)
Bachelor’s	5,138 (23.8)
Less than Bachelor’s	11,863 (54.9)
Annual household income	
≥$100,000	7,742 (35.8)
$50,000-$100,000	7,232 (33.4)
$20,000-$50,000	4,367 (20.2)
<$20,000	987 (4.6)
Refused/do not know	1,305 (6.0)
Diabetes	
None	17,919 (83.6)
Type I	111 (0.5)
Type II	1,851 (8.6)
Suspect/neither type	1,565 (7.3)
Smoking status	
Never	10,124 (47.0)
Former	9,637 (44.7)
Current	1,794 (8.3)
Alcohol intake frequency	
Never	404 (1.9)
Occasional	8,217 (38.0)
Weekly	9,340 (43.2)
Daily	3,662 (16.9)
BMI (kg/m^2^)	28.0 ± 5.3
HDL-C (mmol/L)	1.5 ± 0.5
TC (mmol/L)	5.1 ± 1.1
Log-TG [log(mmol/L)]	0.4 ± 0.5

BMI, body mass index; CLSA, canadian longitudinal study on aging; DBP, diastolic blood pressure; HDL-C, high-density lipoprotein cholesterol; IOP, intraocular pressure; log-TG, natural log-transformed triglycerides; PP, pulse pressure; PRS, polygenic risk score; SBP, systolic blood pressure; SD, standard deviation; TC, total cholesterol

Data presented as mean ± SD for continuous variables or n (%) for categorical variables.

### 3.2 Observational analyses

Separate multivariable linear regression models adjusted for age, sex, province, marital status, education, annual household income, diabetes, smoking status, alcohol intake frequency, BMI, HDL-C, TC, log-TG, and the top ten genetic principal components found SBP, DBP, and PP to each be positively associated with IOP (*P* ≤ 3.82 × 10^−17^; Table 2). Similar results were obtained when using minimal covariate adjustment for age, sex, province, and the top ten genetic principal components (*P* ≤ 4.84 × 10^−23^). SBP, DBP, and PP remained positively associated with IOP after excluding participants taking IOP-lowering or antihypertensive medications (*P* ≤ 9.63 × 10^−18^; Supplementary Table S2). There was no evidence of interactions between SBP, DBP, or PP and the IOP PRS, regardless of covariate adjustment (*P* ≥ 0.122; Table 3) or after excluding participants taking IOP-lowering or antihypertensive medications (*P* ≥ 0.149; Supplementary Table S3).

**TABLE 2:** Linear regression analyses of the association Between BP and IOP.

Exposure	IOP (mmHg)			
	Model 1* (*n* = 21,633)		Model 2^†^ (*n* = 21,125)	
	*β* (95% CI)	*P*-value	*β* (95% CI)	*P*-value
SBP (10 mmHg)	0.232 (0.204, 0.259)	1.56 × 10^−59^	0.220 (0.190, 0.250)	3.58 × 10^−46^
DBP (10 mmHg)	0.403 (0.356, 0.450)	1.27 × 10^−62^	0.370 (0.320, 0.420)	5.04 × 10^−47^
PP (10 mmHg)	0.217 (0.174, 0.260)	4.84 × 10^−23^	0.195 (0.150, 0.240)	3.82 × 10^−17^

BMI, body mass index; BP, blood pressure; DBP, diastolic blood pressure; HDL-C, high-density lipoprotein cholesterol; IOP, intraocular pressure; log-TG, natural log-transformed triglycerides; PP, pulse pressure; SBP, systolic blood pressure; TC, total cholesterol

The Bonferroni-corrected significance threshold was *P* < 0.017 (*α*_Bonferroni_ = 0.05/3).

^*^ Model 1: Adjusted for age, sex, province, and the top ten genetic principal components.

^†^ Model 2: Adjusted for covariates as well as marital status, education, annual household income, diabetes, smoking status, alcohol intake frequency, BMI, HDL-C, TC, and log-TG.

**TABLE 3:** Linear regression interaction analyses of BP and the IOP PRS on IOP.

Analysis	IOP (mmHg)					
	Exposure		IOP PRS		Exposure × IOP PRS	
	*β* (95% CI)	*P*-value	*β* (95% CI)	*P*-value	*β* (95% CI)	*P*-value
Model 1* (*n* = 21,633)						
SBP (10 mmHg)	0.234 (0.206, 0.261)	**9.22 × 10** ^**−63**^	0.498 (0.176, 0.819)	**0.002**	0.018 (−0.007, 0.044)	0.153
DBP (10 mmHg)	0.410 (0.364, 0.457)	**2.45 × 10** ^**−67**^	0.581 (0.238, 0.924)	**9.15 × 10** ^**−4**^	0.020 (−0.025, 0.064)	0.384
PP (10 mmHg)	0.216 (0.174, 0.259)	**1.23 × 10** ^**−23**^	0.588 (0.404, 0.772)	**3.66 × 10** ^**−10**^	0.028 (−0.008, 0.065)	0.126
Model 2^†^ (*n* = 21,125)						
SBP (10 mmHg)	0.221 (0.192, 0.251)	**1.50 × 10** ^**−48**^	0.499 (0.175, 0.822)	**0.003**	0.018 (−0.007, 0.044)	0.159
DBP (10 mmHg)	0.377 (0.328, 0.426)	**1.16 × 10** ^**−50**^	0.582 (0.237, 0.927)	**9.36 × 10** ^**−4**^	0.019 (−0.025, 0.064)	0.394
PP (10 mmHg)	0.193 (0.149, 0.238)	**1.91 × 10** ^**−17**^	0.585 (0.400, 0.770)	**5.74 × 10** ^**−10**^	0.029 (−0.008, 0.066)	0.122

BMI, body mass index; BP, blood pressure; CI, confidence interval; DBP, diastolic blood pressure; HDL-C, high-density lipoprotein cholesterol; IOP, intraocular pressure; log-TG, natural log-transformed triglycerides; PP, pulse pressure; PRS, polygenic risk score; SBP, systolic blood pressure; TC, total cholesterol

P-values that achieved a Bonferroni-corrected significance threshold of *P* < 0.017 (*α*_Bonferroni_ = 0.05/3) are in bold.

^*^ Model 1: Adjusted for age, sex, province, and the top ten genetic principal components.

^†^ Model 2: Adjusted for covariates as well as marital status, education, annual household income, diabetes, smoking status, alcohol intake frequency, BMI, HDL-C, TC, and log-TG.

### 3.3 Genetic instruments for one-sample MR

All PRS instruments for the one-sample MR analyses, including the Steiger-filtered BP PRS instruments, demonstrated sufficient instrument strength (partial *F*-statistics ≥650.83) to avoid potential weak instrument bias (Supplementary Table S4). A few associations between the BP PRS instruments and measured confounders remained significant after Bonferroni correction (*P* < 0.005; Supplementary Tables S5–S7). The primary SBP and DBP PRS instruments were associated with TC (*P* = 2.80 × 10^−4^ and *P* = 1.82 × 10^−5^, respectively), while the primary PP PRS instrument was associated with diabetes (*P* = 0.004). The Steiger-filtered BP PRS instruments showed a similar pattern, with the additional association between the SBP PRS and diabetes (*P* = 0.001). The IOP PRS was only found to have a Bonferroni-corrected significant association with TC (*P* = 4.91 × 10^−3^; Supplementary Table S8).

### 3.4 One-sample MR analyses

One-sample MR using 2SLS regression adjusted for age, sex, province, and the top ten genetic principal components found genetically predicted SBP to be positively associated with IOP (*β* = 0.227 mmHg per 10 mmHg increase in SBP, 95% CI [0.074, 0.381], *P* = 0.004) (Table 4). Neither genetically predicted DBP nor PP were found to be significantly associated with IOP (*P* ≥ 0.205). The 2SLS regression models with IOP as the exposure and the BP traits as the outcome, did not provide evidence to support potential reverse causation (*P* ≥ 0.103). Similar results were obtained when using 2SLS regression with additional adjustment for marital status, education, annual household income, diabetes, smoking status, alcohol intake frequency, BMI, HDL-C, TC, and log-TG. One-sample MR performed using Steiger-filtered BP PRS instruments did not find any genetically predicted BP trait to be significantly associated with IOP, regardless of included covariates (*P* ≥ 0.056; Supplementary Table S9). After excluding participants taking IOP-lowering or antihypertensive medications, genetically predicted SBP was positively associated with IOP at nominal significance in both the minimally adjusted (*β* = 0.292 mmHg per 10 mmHg increase in SBP, 95% CI [0.036, 0.548], *P* = 0.025) and fully adjusted (*β* = 0.284 mmHg per 10 mmHg increase in SBP, 95% CI [0.056, 0.512], *P* = 0.015) models (Supplementary Table S10).

**TABLE 4:** Bidirectional one-sample MR analyses of the associations Between BP and IOP.

Exposure	Outcome	No. of PRS SNPs	Model 1* (*n* = 21,633)		Model 2^†^ (*n* = 21,125)	
			*β* (95% CI)	*P*-value	*β* (95% CI)	*P*-value
SBP (10 mmHg)	IOP (mmHg)	522	0.227 (0.074, 0.381)	**0.004**	0.225 (0.074, 0.376)	**0.004**
DBP (10 mmHg)	IOP (mmHg)	477	0.162 (−0.088, 0.413)	0.205	0.210 (−0.039, 0.458)	0.098
PP (10 mmHg)	IOP (mmHg)	456	0.116 (−0.114, 0.346)	0.322	0.133 (−0.096, 0.361)	0.255
IOP (mmHg)	SBP (mmHg)	122	−0.141 (−0.473, 0.190)	0.404	−0.108 (−0.419, 0.203)	0.494
IOP (mmHg)	DBP (mmHg)	122	−0.163 (−0.360, 0.033)	0.103	−0.143 (−0.331, 0.044)	0.134
IOP (mmHg)	PP (mmHg)	122	0.022 (−0.192, 0.236)	0.838	0.035 (−0.172, 0.241)	0.740

BMI, body mass index; BP, blood pressure; CI, confidence interval; DBP, diastolic blood pressure; HDL-C, high-density lipoprotein cholesterol; IOP, intraocular pressure; log-TG, natural log-transformed triglycerides; MR, mendelian randomization; PP, pulse pressure; PRS, polygenic risk score; SBP, systolic blood pressure; SNP, single nucleotide polymorphism; TC, total cholesterol P-values that achieved a Bonferroni-corrected significance threshold of *P* < 8.33 × 10−3. (*α*_Bonferroni_ = 0.05/6) are in bold.

^*^ Model 1: Adjusted for age, sex, province, and the top ten genetic principal components.

^†^ Model 2: Adjusted for covariates, marital status, education, annual household income, diabetes, smoking status, alcohol intake frequency, BMI, HDL-C, TC, and log-TG.

### 3.5 Two-sample MR analyses

The primary two-sample MR analysis using IVW-MRE found genetically predicted SBP to be positively associated with IOP (*β* = 0.216 mmHg per 10 mmHg increase in SBP, 95% CI [0.043, 0.390], *P* = 0.015; Table 5). The MR-ConMix (*β* = 0.314 mmHg per 10 mmHg increase in SBP, 95% CI [0.134, 0.565], *P* = 0.001) and MR-PRESSO (*β* = 0.239 mmHg per 10 mmHg increase in SBP, 95% CI [0.075, 0.402], *P* = 0.004) methods also found genetically predicted SBP to be significantly associated with IOP. Neither the IVW-MRE nor the robust MR methods found genetically predicted DBP or PP to be significantly associated with IOP.

**TABLE 5:** Two-sample MR analyses of the association Between BP and IOP.

Exposure	MR method	No. of SNPs	IOP (mmHg)	
			*β* (95% CI)	*P*-value
SBP (10 mmHg)	IVW-MRE	522	0.216 (0.043, 0.390)	**0.015**
	MR-egger	522	−0.175 (−0.601, 0.251)	0.421
	Weighted median	522	0.153 (−0.082, 0.389)	0.201
	Weighted mode	522	−0.158 (−1.224, 0.908)	0.772
	MR-ConMix	522	0.314 (0.134, 0.565)	**0.001**
	MR-PRESSO	515	0.239 (0.075, 0.402)	**0.004**
DBP (10 mmHg)	IVW-MRE	477	0.154 (−0.133, 0.440)	0.293
	MR-egger	477	0.074 (−0.629, 0.776)	0.837
	Weighted median	477	0.187 (−0.220, 0.595)	0.368
	Weighted mode	477	0.052 (−1.550, 1.654)	0.949
	MR-ConMix	477	0.216 (−0.187, 0.577)	0.244
	MR-PRESSO	474	0.130 (−0.148, 0.409)	0.359
PP (10 mmHg)	IVW-MRE	456	0.100 (−0.161, 0.360)	0.454
	MR-egger	456	0.134 (−0.469, 0.738)	0.663
	Weighted median	456	−0.014 (−0.384, 0.356)	0.942
	Weighted mode	456	−0.203 (−1.928, 1.522)	0.818
	MR-ConMix	456	−0.092 (−0.449, 0.322)	0.664
	MR-PRESSO	451	0.149 (−0.098, 0.396)	0.239

BP, blood pressure; CI, confidence interval; DBP, diastolic blood pressure; IOP, intraocular pressure; IVW-MRE, inverse-variance weighted multiplicative random-effects; MR, mendelian randomization; MR-ConMix, MR, contamination mixture; MR-PRESSO, MR, Pleiotropy RESidual Sum and Outlier; PP, pulse pressure; SBP, systolic blood pressure; SNP, single nucleotide polymorphism

*P*-values that achieved a Bonferroni-corrected significance threshold of *P* < 0.017 (*α*_Bonferroni_ = 0.05/3) are in bold.

Diagnostic metrics for the two-sample MR analyses are presented in Supplementary Table S11. Significant heterogeneity in SNP-specific causal effect estimates was observed for all BP traits (*I* ^2^ ≥ 30.5% and Cochran’s *Q* statistic *P*-value ≤1.04 × 10^−9^). Accounting for potential directional pleiotropy using MR-Egger did not eliminate this heterogeneity (Rücker’s *Q*′ statistic *P*-value ≤8.72 × 10^−10^). The MR-PRESSO global test found evidence of overall horizontal pleiotropy for all BP SNP instruments (global test *P*-value <0.001). The MR-Egger intercept test only found evidence of directional pleiotropy for the SBP SNP instruments (intercept *P*-value = 0.049). Potential regression dilution bias was found to have a minimal impact on the MR-Egger analyses ( $I_{GX}^{2}$ ≥ 92.3%). Finally, the MR-ConMix method defined 78.2%, 77.8%, and 77.6% of the SBP, DBP, and PP SNP instruments to be valid genetic instruments, respectively.

Two-sample MR analyses using Steiger-filtered SNPs provided nominally significant evidence of genetically predicted SBP being positively associated with IOP when using the MR-ConMix method (*β* = 0.470 mmHg per 10 mmHg increase in SBP, 95% CI [0.042, 0.702], *P* = 0.032; Supplementary Table S12). It was not possible to conduct MR-PRESSO using the Steiger-filtered SNPs since there were no outlier SNPs identified for removal. There was no evidence of significant heterogeneity, directional pleiotropy, or regression dilution bias (Supplementary Table S11). The MR-ConMix method defined 86.0%, 94.1%, and 95.7% of the SBP, DBP, and PP SNP instruments after Steiger filtering to be valid genetic instruments, respectively.

Two-sample MR analyses using SNP-IOP estimates from the CLSA that excluded participants taking IOP-lowering medications (Supplementary Table S13) corroborated our findings from the primary two-sample MR analyses. The IVW-MRE (*β* = 0.238 mmHg per 10 mmHg increase in SBP, 95% CI [0.080, 0.396], *P* = 0.003), MR-ConMix (*β* = 0.358 mmHg per 10 mmHg increase in SBP, 95% CI [0.109, 0.593], *P* = 0.006), and MR-PRESSO (*β* = 0.251 mmHg per 10 mmHg increase in SBP, 95% CI [0.095, 0.407], *P* = 0.002) methods found genetically predicted SBP to be positively associated with IOP at Bonferroni-corrected significance. Unlike the primary analysis, the MR-Egger intercept test did not provide evidence of directional pleiotropy for SBP (intercept *P*-value = 0.062; Supplementary Table S14).

## 4 Discussion

The goal of this study was to investigate the association of BP with IOP, assess potential causality, and evaluate potential interactions between BP and genetic susceptibility to elevated IOP. Multivariable linear regression found SBP, DBP, and PP to be positively associated with IOP. There was no evidence that the associations of SBP, DBP, or PP with IOP differed according to genetic susceptibility to elevated IOP. The one-sample and two-sample MR analyses only found genetically predicted SBP to be positively associated with IOP. Individual-level analyses that excluded participants taking IOP-lowering or antihypertensive medications corroborated our observational and one-sample MR results. Results from two-sample MR analyses that used SNP-IOP association estimates from the CLSA after excluding participants taking IOP-lowering medications were consistent with the results from our primary two-sample MR analyses. The use of Steiger-filtered SNPs provided some support for the two-sample MR findings, but not the one-sample MR findings.

When conducting an MR analysis, it is essential to evaluate the validity of the underlying assumptions for the genetic instruments and assess the impact of potential violations (Davies et al., 2018). The presence of significant associations between our one-sample MR PRS instruments and measured confounders would most likely indicate violations of the IV Independence Assumption (Carter and Anderson, 2024). However, the conclusions from our one-sample MR analysis did not drastically change after adjusting our 2SLS regression models with additional covariates. This suggests that the effects of the genetic instruments on potential confounders were minimal in comparison to their effects on their intended exposures (Davies et al., 2018).

For our two-sample MR analysis, multiple methods were employed to take advantage of differences in their underlying assumptions (Slob and Burgess, 2020). While the IVW method offers the most statistical power for two-sample MR, it assumes that all genetic instruments are valid. Robust MR methods rely on less stringent assumptions and can be used to obtain consistent results, even when there are violations of the core IV assumptions. The detection of directional pleiotropy in our two-sample MR analysis of SBP and IOP raised concern about bias in the IVW-MRE estimate. However, robust MR methods (MR-ConMix and MR-PRESSO) provided corroborating evidence of the positive association between genetically predicted SBP and IOP being statistically significant. Consistent results across multiple methods relying on different underlying assumptions provides stronger evidence than findings from a limited number of tested methods.

Observational analyses have generally shown SBP, DBP, and PP to be positively associated with IOP (Bae et al., 2014; Zhao et al., 2014; Chan et al., 2016; Han et al., 2018; Plotnikov et al., 2022; Yasukawa et al., 2022). The results from our multivariable linear regression analysis were consistent with these findings. In fact, our association estimates for SBP and DBP are comparable with those from the Zhao et al. (2014) meta-analysis. Our one-sample and two-sample MR results found some evidence of a potential causal effect of SBP on IOP. Discrepancies between our findings and those of previous BP-IOP MR studies could be attributed to differences in IV selection and statistical methods (Deng et al., 2020; Slob and Burgess, 2020; Burgess et al., 2024). The previous MR studies obtained their SNP-BP associations from smaller GWASs and performed LD clumping using more lenient *r* ^2^ values to identify independent SNPs as potential genetic instruments for their main analyses (Plotnikov et al., 2022; Tan et al., 2022; Rajasundaram et al., 2024).

Non-significant results from our one-sample and two-sample MR analyses could be a consequence of insufficient statistical power. Consider our primary one-sample MR analysis assessing potential causal effects of BP on IOP, with minimal covariate adjustment (age, sex, province, and the top ten genetic principal components). We used the mRnd online web tool (https://shiny.cnsgenomics.com/mRnd/) to investigate statistical power (Brion et al., 2013). Assuming a type I error rate of α = 0.05, we had 80% power to detect causal effect estimates (expressed as the mmHg change in IOP per 10 mmHg increase for a specific BP trait) of at least 0.219, 0.358, and 0.329 for SBP, DBP, and PP, respectively. The minimum detectable effect estimates for DBP and PP are larger than our reported causal effect estimates.

This study has several strengths. To our knowledge, it is the first to use a one-sample MR design to investigate the causal effects of BP on IOP and whether these effects are modified by genetic susceptibility to elevated IOP (Liu et al., 2022; Plotnikov et al., 2022; Tan et al., 2022; Julian et al., 2023; Wang K. et al., 2023; Rajasundaram et al., 2024). A reverse one-sample MR analysis was also performed to assess reverse causation. MR analyses leveraged summary statistics from the largest BP GWAS meta-analysis available to date (Keaton et al., 2024). Finally, the availability of detailed phenotypic and genetic data in the CLSA enabled a comprehensive investigation utilizing both one-sample and two-sample MR approaches. CLSA participants were independent of those included in the BP (Keaton et al., 2024) and IOP (Khawaja et al., 2018) GWAS meta-analyses, eliminating concerns regarding bias arising from participant overlap (Burgess and Thompson, 2013; Burgess et al., 2016).

Despite these strengths, there are several limitations that should be acknowledged. First, the relationship between BP and IOP was investigated using a cross-sectional design. These dynamic traits vary within individuals and over time (Liu et al., 2003; Hartmann et al., 2024). A bidirectional MR approach was used to investigate causality and establish temporality (Boehm and Zhou, 2022). Second, restricting the analysis to individuals of European descent limits the generalizability of the findings. This restriction was necessary to minimize bias from population stratification (Carter and Anderson, 2024). Out of 26,622 CLSA Comprehensive Cohort participants with genotype data, only 1,967 (7.4%) were of non-European descent (Forgetta et al., 2022). Finally, the SNP-IOP association estimates derived from the CLSA were likely to be less precise than estimates that could be obtained from larger biobanks. This would impact the statistical power for two-sample MR (Deng et al., 2020) and Steiger filtering (Hemani et al., 2017).

This study found SBP, DBP, and PP to be positively associated with IOP. Genetic susceptibility to elevated IOP did not appear to influence these associations. One-sample and two-sample MR analyses provided evidence supporting a potential causal effect of SBP on IOP. Elevated BP could potentially increase IOP by either increasing the production of aqueous humor through ultrafiltration at the ciliary body or reducing aqueous humor outflow through increased resistance resulting from elevated episcleral venous pressure (Bulpitt et al., 1975; Shiose, 1984). These mechanisms may be driven more by SBP than DBP, which could explain our lack of MR evidence supporting a causal effect of DBP on IOP. Future work should focus on trying to understand how the genetic architecture of glaucoma and its endophenotypes could influence the effect of BP on IOP. If confirmed, a causal effect of BP on IOP could lead ophthalmologists treating elevated IOP to encourage their patients to also have their BP under control.

## Data Availability

The data analyzed in this study is subject to the following licenses/restrictions: Data are available from the Canadian Longitudinal Study on Aging (www.clsa-elcv.ca) for researchers who meet the criteria for access to de-identified CLSA data. Requests to access these datasets should be directed to www.clsa-elcv.ca. Code is made available on GitHub (https://github.com/Roy-Gagnon-lab/Bastasic2_2025).
